# SOCIAL HEALTH, MOBILITY, AND TECHNOLOGY: ACCESSIBILITY WITHIN AGE-FRIENDLY COMMUNITIES

**DOI:** 10.1093/geroni/igz038.2079

**Published:** 2019-11-08

**Authors:** Haley B Gallo, Lia W Marshall, Lené Levy-Storms, Kathleen H Wilber, Anastasia Loukaitou-Sideris

**Affiliations:** 1 Leonard Davis School of Gerontology, University of Southern California, Los Angeles, California, United States; 2 Department of Social Welfare, University of California Los Angeles, Los Angeles, California, United States; 3 Department of Social Welfare, University of California Los Angeles, Department of Medicine/Geriatrics, University of California Los Angeles, Los Angeles, California, United States; 4 Department of Urban Planning, University of California Los Angeles, Los Angeles, California, United States

## Abstract

To explore how access to transportation and technology/social media influence social connectivity among an ethnically diverse group of vulnerable low-income older adults, six focus groups were conducted (N=48) in English, Spanish, and Korean at a senior services agency. Qualitative thematic analyses revealed overarching themes that fit within the World Health Organization’s Age-Friendly Domains of Livability. The sub-theme “barriers and facilitators to accessibility” ran through each of the overarching themes, demonstrating how specific factors of the built, social, and community health environments intersect to promote or hinder social connection. Although transportation and technology uses were linked to social engagement, challenges with the built environment and limited financial resources hindered older adults’ abilities to remain engaged in their communities, both in-person and electronically. Age-Friendly initiatives must continue to consider the community-specific barriers and facilitators for older adults to remain physically and socially connected to the community.

